# Mechanical Behavior of Melt-Mixed 3D Hierarchical Graphene/Polypropylene Nanocomposites

**DOI:** 10.3390/polym12061309

**Published:** 2020-06-08

**Authors:** Karolina Gaska, Georgia C. Manika, Thomas Gkourmpis, Davide Tranchida, Antonis Gitsas, Roland Kádár

**Affiliations:** 1 Department of Industrial and Materials Science, Division of Engineering Materials, Chalmers University of Technology, SE-412 96 Gothenburg, Sweden; georgia.manika@chalmers.se (G.C.M.); roland.kadar@chalmers.se (R.K.); 2 Innovation & Technology, Borealis AB, SE-444 86 Stenungsund, Sweden; thomas.gkourmpis@borealisgroup.com; 3 Innovation & Technology, Borealis Polyolefine GmbH, St.-Peter-Straße 25, 4021 Linz, Austria; davide.tranchida@borealisgroup.com (D.T.); antonis.gitsas@borealisgroup.com (A.G.)

**Keywords:** graphene, nanocomposites, mechanical properties, time–temperature superposition

## Abstract

The mechanical properties of novel low percolation melt-mixed 3D hierarchical graphene/polypropylene nanocomposites are analyzed in this study. The analysis spans a broad range of techniques and time scales, from impact to tensile, dynamic mechanical behavior, and creep. The applicability of the time–temperature superposition principle and its limitations in the construction of the master curve for the isotactic polypropylene (iPP)-based graphene nanocomposites has been verified and presented. The Williams–Landel–Ferry method has been used to evaluate the dynamics and also Cole–Cole curves were presented to verify the thermorheological character of the nanocomposites. Short term (quasi-static) tensile tests, creep, and impact strength measurements were used to evaluate the load transfer efficiency. A significant increase of Young’s modulus with increasing filler content indicates reasonably good dispersion and adhesion between the iPP and the filler. The Young’s modulus results were compared with predicted modulus values using Halpin–Tsai model. An increase in brittleness resulting in lower impact strength values has also been recorded.

## 1. Introduction

Over the last 15 years, the performance of high aspect ratio fillers has shown great promise and opened a wide range of opportunities in the area of polymer nanocomposites [1]. Consequently the use of different fillers of high-aspect ratio has led to the development of high-performance and multifunctional materials with a wide range of industrial applications, for example, as gas-barrier membranes [2,3,4,5], antibacterial surfaces [6,7,8], heat-sinks [9,10] and electronics [11].

Ever since its discovery [12], graphene is one of the most widely studied high aspect ratio fillers, due to the reportedly exceptional electrical, mechanical and thermal properties [13,14,15,16]. Consequently, graphene/polyolefin nanocomposites have attracted significant attention both in academia and industry [13,16,17]. One of the biggest challenges in polymers and polymer-based systems, including nanocomposites, is that their mechanical response depends strongly on the testing time scale, due to the viscoelastic nature of such materials.

The introduction of a graphene into a polymer matrix has been reported to enhance the mechanical performance, as this is seen, for example, through the Young’s modulus [18,19]. However, this enhancement depends on properties of the filler, namely its size, shape, aspect ratio, content, as well as processing conditions [20]. Three main aspects with respect to the load transfer mechanisms are important for the mechanical properties of polymer nanocomposites [21]: (i) filler aspect ratio, (ii) compatibility with the polymer matrix, and (iii) influence of the filler on the crystalline-amorphous structure. Many researchers focus mainly on investigating mechanical properties using tensile testing or impact strength usually at room temperature [15,17]. As an example, Yang et al. incorporated functionalized reduced graphene oxide into polypropylene by solution mixing and obtained an improvement in Young’s modulus and tensile strength [22]. Todd and Bielawski used in situ polymerization to disperse reduced graphene oxide in polyethylene and obtained 170% of increase in tensile modulus for sample with 5.2 wt.% [23]. In contrast, Song et al. achieved a 75% increase in yield strength and 74% increase in the Young’s modulus of polypropylene (PP) by the addition of only 0.42 vol.% of reduced graphene oxide [24]. A considerable amount of literature has been published on mechanical properties of PP/graphene nanocomposites, however usually the used production method is challenging to scale-up [23,24,25,26]. Therefore, in this work, we choose a melt mixing method as a promising, economical technique that can be used for large-scale production [27].

The long-term exposure of polymer nanocomposites to load cycles, pressure changes or temperature fluctuations has the potential to lead to gradual change of the initial properties of the material [28]. Therefore, the prediction of long-term viscoelastic properties of such systems is very important and crucial for their proper application and further safe operation. The importance of long-term properties of polymeric materials cannot be understated from both practical and fundamental points of view due to the viscoelastic nature of polymeric materials [29]. Creep control is an essential feature for the lifetime design of polymer parts while, particularly in high temperature environments, creep can have a severe effect on component performance [30]. From the fundamental point of view, creep tests minimize measurement issues related to the influence of experimental conditions (e.g., loading rate) common in short-term, e.g., tensile tests [31].

The time–temperature superposition principle (TTS) is a suitable method to predict the viscoelastic properties of polymers and polymer matrix composites [32]. Experimentally determined frequency-dependent curves of isothermal dynamic modulus, might be used to build so called master curves simulating long-term viscoelastic behavior. In addition, such master curves can expand the range of measured frequency, especially with respect to damping properties at frequencies otherwise difficult to directly determine experimentally. The modulus of a viscoelastic material is known to be time dependent at a constant temperature and temperature dependent at a constant time [33,34]. Therefore, obtained isothermal curves of moduli vs. frequency can be horizontally shifted in reference to a chosen temperature building a master curve. Moreover, the relation between shift factors of all curves versus temperatures can be described by mathematical models like for example the Williams–Landel–Ferry (WLF) [35] and Arrhenius equation for secondary relaxations. However, it should be noted that TTS was originally dedicated for amorphous polymers, which are considered as thermorheologically simple materials [33,36]. The complex thermal behavior of some semi-crystalline materials might remain challenging in the case of applicability of TTS. Some authors suggested that more complex materials’ master curves can be constructed by combining horizontal and vertical shifts [37,38,39]. In case of polypropylene many researchers have used Arrhenius equation to validate the TTS method [36,40].

Polyolefins are the most commonly used polymers today due to their versatility in terms of performance and relatively simple preparation methods. The latter allows for the production of large amounts of polymer in a streamlined manner while keeping the consistency of the final product as stable as possible, thus leading to a significantly reduced overall cost [41]. Polypropylene is one of the most commonly used polyolefins with a wide range of applications like medical devices, automotive parts, or pressure pipes [42]. Traditionally, isotactic polypropylene (iPP) is produced via a Ziegler–Natta or Metalocene-type catalysts [43]. Various advances in catalyst and reactor design during the last 50 years have allowed for the development of a wide range of polypropylenes with unique characteristics that can tackle most of the limitations of the material, like high molding shrinkage, low stiffness or poor impact toughness [44,45,46]. The abovementioned limitations can be overcome by the addition of fillers like talc [47], calcium carbonate [48], carbon fibers [49], glass fibers [50], or high aspect ratio fillers like graphene [51] and carbon nanotubes [52]. Due to its technological importance, a number of studies on the mechanical performance of PP/graphene nanocomposites has been made. The majority of these studies has been based on graphene nanoplatelets (GNP). Despite the improvements in electrical [53], thermal [54], and mechanical [55] properties reported for these types of filler, they still suffer from relatively poor dispersion and significant agglomeration [1]. Since the reinforcement of the nanocomposite is intimately related with the aspect ratio and the interfacial area between filler and matrix [25], it is expected that improved dispersion can lead to an overall improvement in mechanical properties.

In this work, we present an industrially relevant system of polypropylene with a novel hierarchical reduced graphene oxide nanostructure (HrGO), that is capable of achieving superior levels of dispersion as seen through the observed electrical percolation threshold [27]. Previously we have seen that the efficient level of dispersion allows us access to the primary filler particle, and in this work, we are focusing on studying in more detail the all-important mechanical properties of these systems at different time scales. While the electrical percolation stands out as proof of the superior dispersion achievable and the overall potential of the filler, there are fundamental questions regarding the mechanical performance of the iPP-HrGO composites that need to be addressed. Several competing mechanisms that could enhance or negatively influence the mechanical properties can be envisioned. Firstly, polyolefins are inherently incompatible with graphene-type fillers, which typically results in poor dispersion and reduced interfacial strength [18,43]. While the dispersion quality has been thoroughly assessed [49], questions remain regarding the transfer load efficiency between the HrGO filler and the iPP matrix. Furthermore, due to the 3D hierarchical filler morphology and subsequent filler distortions during the preparation stage, the ability to access the primary graphene particle from mechanical point of view needs to be tested. The filler has also been shown to have strong nucleation effects [27,56], resulting in an alteration of the crystalline structure, that can have a significant impact on the mechanics of the system. In this context, we focus on dynamic, tensile, impact, and creep properties of the novel 3D hierarchical reduced graphene oxide. This paper focuses on studying a few basic mechanical properties of these nanocomposites at different reference time scales, spanning from short term to long term behavior. Through DMTA tests, we aim in this study to verify the applicability of the time–temperature superposition principle in construction of the master curve for the PP-based graphene nanocomposites. Short term tensile stress–strain and creep tests are evaluated as measures of the load transfer efficiency. The impact strength is also estimated. Overall, the results can help with understanding the use of the novel types on nanocomposites for e.g., in structural applications.

## 2. Materials and Methods

**Figure 1:**
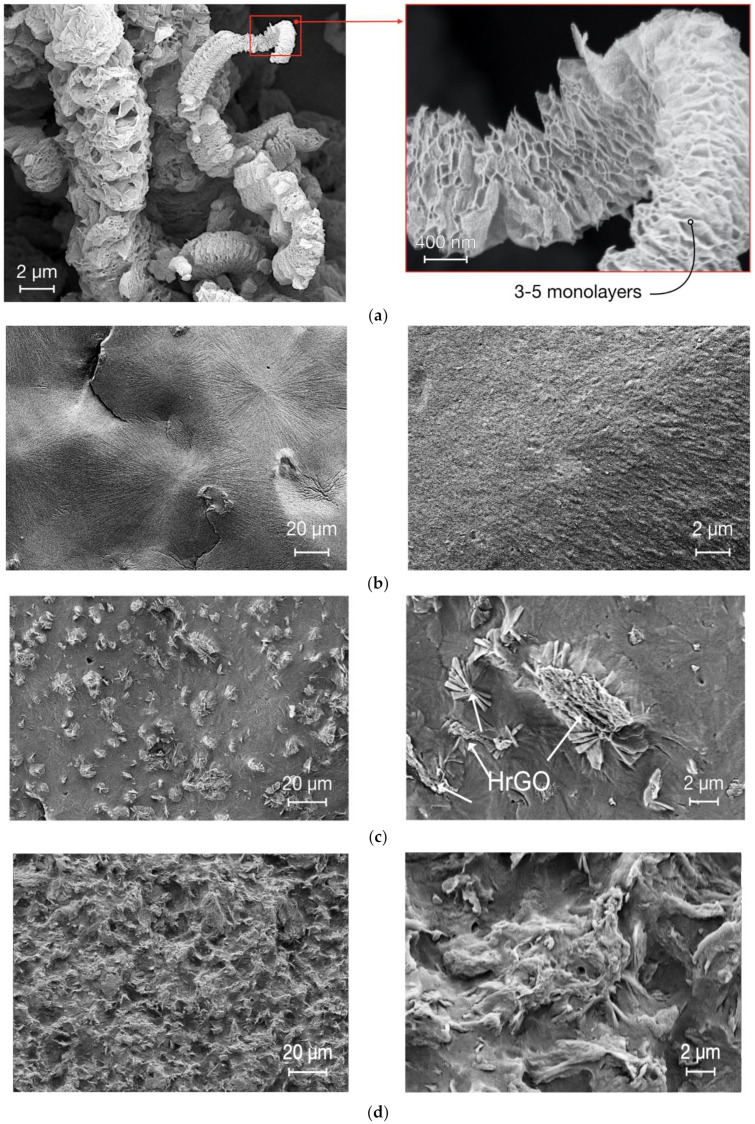
Morphology via scanning electron microscopy (SEM) for (**a**) the 3D hierarchical reduced graphene oxide (HrGO) in powder form, (**b**) the polypropylene (iPP) matrix (**c**) iPP-HrGO with filler loading of 2 wt.% and (**d**) 4 wt.%.

A highly isotactic (>90%) polypropylene (iPP) obtained from Borealis AG (Vienna, Austria) was used. The iPP has molecular weight 300 kg/mol, and dispersity index, *Ð* = 8. De-agglomerated hierarchical thermally reduced graphene oxide supplied by Cabot Corporation (Boston, MA, USA) has been used. In Figure 1a, SEM imaging of the fillers and their hierarchical structure can be seen.

### 2.1. Sample Preparation

All nanocomposites were prepared using a Brabender mixer Type W50 (Brabender GmbH & Co. KG, Duisburg, Germany) driven by a Brabender Plasticorder (Brabender GmbH & Co. KG, Duisburg, Germany) initially melting the iPP at 210 °C at 20 rpm for 15 min following with mixing with graphene at 50 rpm for 15 min. After mixing, the samples were compression molded to obtain dimensions as follows: (100 × 100 × 1.5) mm^3^. The temperature maintained during the process was 200 °C at 50 bar for 5 min, the process was terminated after cooling the press to room temperature at ≈10 °C min^−1^ while maintaining the pressure. It is worth mentioning that the melt-mixing process was kept as simple as possible and there was no pre-treatment of the filler or pre- and post- mixing stages in order to obtain more efficient filler distribution. The production process was kept as similar as possible to large-scale industrial procedures.

### 2.2. Scanning Electron Microscopy

The morphology of nanocomposites was evaluated by means of Scanning Electron Microscopy (SEM) FEI Quanta 200 (FEI Company, Hillsboro, Oregon, USA). All the samples’ surfaces were etched for 1 h using a solution of 1 wt.% potassium permanganate mixed with 86% ortho-phosphoric acid. 5 nm thick Pd-Au layer was deposited onto the observed surfaces prior the SEM imaging.

### 2.3. Dynamic Mechanical Thermal Analysis (DMTA)

Frequency sweeps were performed on an Anton Paar MCR702 TwinDrive rheometer (Graz, Austria) in torsion using a Solid rectangular fixture SRF12 in single drive mode. The temperature was controlled using a convection oven (CTD450TD). Frequency tests were performed in a frequency range of 0.1–40 Hz and the strain amplitude 0.01% in the temperature range −50–150 °C. The storage modulus, loss modulus, and tan δ values were collected during the runs and were plotted versus the frequency. Used samples dimensions were (50 × 10 × 1.5) mm^3^.

### 2.4. Creep Tests

**Figure 2:**
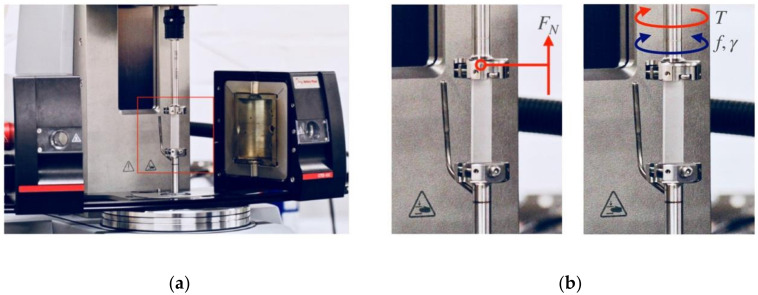
Experimental setups based on the Anton Paar MCR702 rotational rheometer SRF 12 fixture for: (**a**) creep measurements (note: creep sample not represented to scale) and (**b**) dynamic mechanical thermal analysis (DMTA). Both (**a**,**b**) configurations are depicted in open convection oven mode.

Creep measurements were carried out using the same Anton Paar MCR702 TwinDrive rheometer (Graz, Austria) with identical drive, temperature and fixture configurations as described for the DMTA tests (Section 2.3), see Figure 2b. The normal force transducer of the rheometer (max. 50 N) was used to apply a constant tensile stress of 3.5 MPa on the samples at a constant temperature of 120 °C. The applied stress is below 15% of the tensile strength and thus in the linear viscoelastic regime [57]. The sample dimensions were (30 × 4 × 1.5) mm^3^, with each clamp being 7 mm in length. The maximum displacement achievable in the creep tests was limited by the oven cavity size to 30 mm. Due to this limitation, only the primary and secondary creep stages can be evidenced [30].

### 2.5. Short-Term Tensile Tests

Tensile tests have been performed according to ISO 527-2 5A with a pre-load speed of 2 mm/min (2 N pre-load), an initial speed 0.5 mm/min for determining Young’s modulus, followed by an increase to 50 mm/min. The tests were performed on a Zwick Z030 tensile tester (ZwickRoell GmbH & Co. KG, Duisburg, Germany) equipped with a 1 kN load cell and a Zwick Multisense extensometer (ZwickRoell GmbH & Co. KG, Duisburg, Germany). The gripping distance was 50 mm and the gauge length 25 mm. All the measurements were carried out at room temperature (23 °C) and the average values of at least three replicated measurements are considered as final results.

### 2.6. Izod Impact Tests

Impact tests were carried out according to ISO 180 using Izod impact tester Tinius Olsen model 104 (Tinius Olsen Inc. Horsham, Pennsylvania, USA). The measurements were performed at room temperature and obtained values are average values from five replicated measurements for each composite.

## 3. Results and Discussion

### 3.1. Morphology

**Figure 3:**
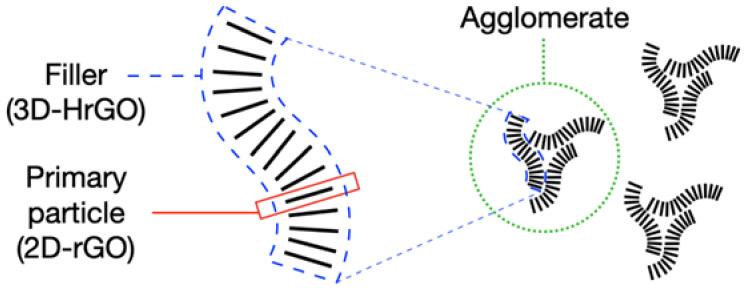
Schematic illustration of the three structural levels that comprise the morphology of HrGO nanocomposites: primary particle, filler and agglomerate.

SEM imaging of pure iPP and iPP with 2 and 4 wt.% of HrGO are shown in Figure 1b–d, respectively. Several further examples of morphology with different filler content can be found in Gkourmpis et al. [27]. When discussing the morphology of the HrGO-iPP nanocomposites, three structural levels need to be considered: (i) the primary particle, i.e., the two-dimensional rGO that make up the HrGO structure, (ii) the filler, i.e., the rGO stack as observed in the powder form, and (iii) agglomerates, i.e., clusters of fillers formed at the incorporation of the filler into the iPP matrix. In Figure 3, a schematic of these structural levels can be seen. The SEM images confirm that there is a reasonably good dispersion of the filler, although, a level of agglomeration is still present. The efficiency of the dispersion has been seen via the excessively low electrical percolation threshold, especially when this is compared with similar systems that are prepared with similar mixing procedures [27]. In addition, a significant distortion and breakage of the fillers is evident, which could also result in the detachment of primary particles. However, based on the electrical percolation threshold we can assert that we have access to the primary particles even though this is difficult to observe through SEM imaging. Figure 1b also shows the typical spherulites observed for the crystallization of non-nucleated iPP. These superstructures are not visible in Figure 1c, following the nucleation activity of the HrGO.

### 3.2. Dynamic Mechanical Thermal Analysis (DMTA)

#### 3.2.1. Construction of the Master Curve for iPP-Graphene

**Figure 4:**
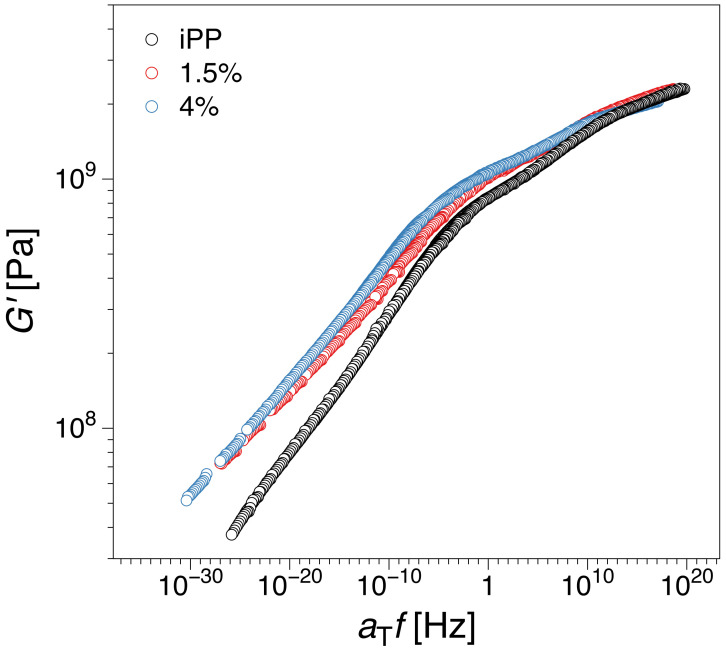
Mastercurve of shear storage modulus, *G′*, obtained by horizontal shifting in frequency for iPP and compositions with 1.5 and 4 wt.% filler content respectively. Temperature range: −50 to 150 °C (223.15 to 423.15 K)

Figure 4 presents master curves of shear storage modulus, *G′*, constructed by horizontal shifts (i.e., only in frequency) of curves measured at different temperatures at a reference temperature of $T_{r}=$ 25 °C. The graphs showing *G′* versus frequency for all temperatures are available Supplementary Figure S1. The storage modulus increased with the content of graphene in comparison to iPP over the entire frequency range, as expected. For a given composition, also expected, the decrease in storage modulus is registered with the decrease of the applied frequency (increase of time) which can be used as a qualitative way of predicting of long-term properties of the material. The frequency range 10^15^ Hz–10^20^ Hz is beyond what is currently experimentally accessible, and therefore, very challenging to interpret from the material’s behavior point of view.

**Figure 5:**
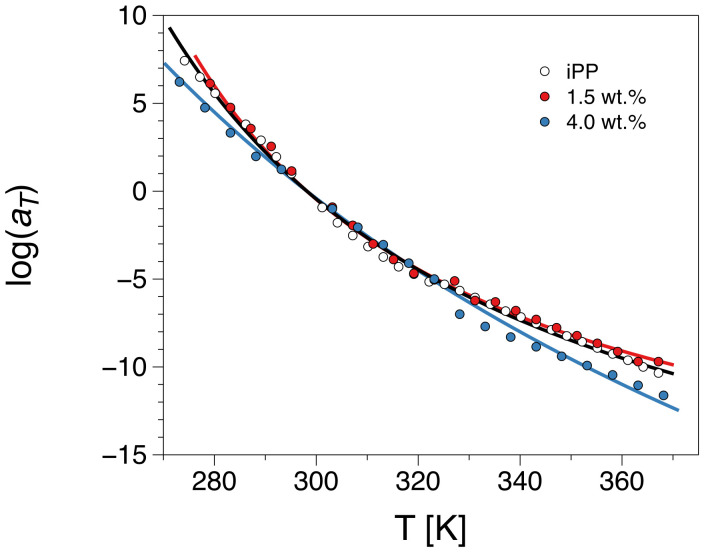
Shift factor, $a_{T}$, versus absolute temperature for the polypropylene (iPP) matrix and iPP-HrGO with 1.5 and 4 wt.% filler loading. The line is a result of a fit to the WLF equation, Equation (1).

The logarithm of the horizontal shift factors $a_{T}$ used for constructing the master curves plotted against the absolute temperature is presented in Figure 5 in the range between 273.15 and 367.15 K, i.e., about 100 K above the glass transition temperature, *T_g_*, of those systems. Their non-linear temperature dependence corresponds to the dynamic arrest occurring while approaching the glass transition, if the same set of data is analyzed in the frequency domain. Various theories have attempted to interpret the slowing-down of the dynamics around the glass transition [58]. Common to most of these theories is some sort of synergistic and density-dependent entropic development, where the relaxation times are effectively dependent on temperature and pressure. As a result, this dynamic change cannot be parameterized by an Arrhenius law. In other words, there is no single activation energy such as in the case of local motions. The Williams–Landel–Ferry (WLF) equation has been thus used to describe the temperature dependence of the shift factors around the glass transition temperature:

(1) $$log\left(a_{T}\right)=\frac{-C_{1}^{r}\left(T-T_{r}\right)}{\left(C_{2}^{r}+\left(T-T_{r}\right)\right)},$$

where $c_{1}^{r}$ and $c_{2}^{r}$ are the WLF parameters at the reference temperature. The values of those parameters with respect to the *T_g_* can be recalculated as: $c_{1}^{g}=\frac{c_{1}^{r}c_{2}^{r}}{c_{2}^{r}+T_{g}-T_{r}}$ and $c_{2}^{g}=c_{2}^{r}+\left(T_{g}-T_{r}\right)$. In fact, the product $c_{1}^{g}\cdot c_{2}^{g}$ can be shown to be equivalent to the ‘activation parameter B’ of the Vogel–Fulcher–Tammann equation if it is written in the form $\tau_{max}=\tau_{0}+\exp\left[\frac{B}{T_{g}-T_{r}}\right]$. Table 1 presents the above mentioned WLF parameters obtained from applying the WLF equation, Equation (1). The glass transition temperature used for the calculations was taken from [27]. As presented in Table 1, the WLF parameters remain approximately similar and within the experimental error for the pure iPP and the 1.5 wt.% composite. Upon the transition to the 4 wt.% loading, the temperature dependence is even stronger. This can be associated with the morphological changes induced by the introduction of the filler and the subsequent nucleation and polymorphism changes [27]. Furthermore, once the filler is introduced in the matrix, the observed nucleation can lead to partial immobilization of the chains in its vicinity. This corresponds to an increase in the slowing-down of the dynamics attributed to an interfacial polypropylene layer formed around the fillers. This layer, even if of limited volume, is having much slower local dynamics than the bulk polypropylene. This immobilization can be associated with the slight shift of the glass transition into higher temperatures [27] and the increase of the activation energy observed in this work.

Here, we must note that the TTS analysis has been developed for thermorheologically simple materials, therefore it is expected that deviations for more complicated systems will be present [59]. The extension of the concept of thermorheologically simple fluid in the case of a semicrystalline system like the one used in this study can be only in a semiempirical manner. Since our system has a significant degree of complexity initiated by the semicrystalline nature, the changes in morphology and the presence of the filler, the validity of the analysis presented here must be seen within these limitations.

#### 3.2.2. Cole-Cole Plots Analysis

**Figure 6:**
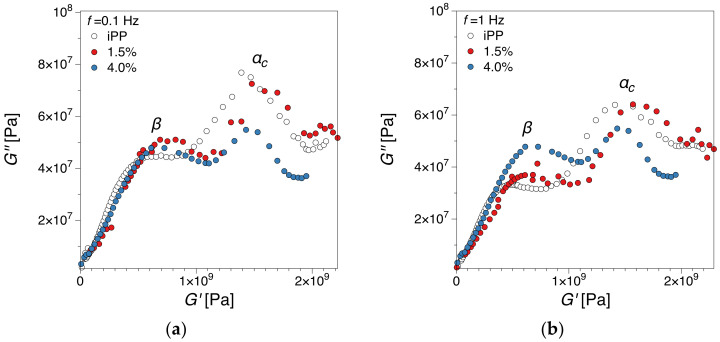
Cole-Cole plots for iPP, and iPP with 1.5 and 4 wt.% of HrGO at (**a**) 0.1 Hz, (**b**) 1 Hz, Temperature decreases from left to right on the graphs.

Cole–Cole plots are defined as an indicator commonly used to verify the thermorheological character of the materials [60]. Cole–Cole plots have been reported to be more sensitive and able to provide additional insight compared to TTS for studying viscoelastic behavior [60]. Since TTS is a method that utilizes the viscoelastic results through the frequency sweep experiments at various temperatures, the accuracy of this determination is strongly affected by the chosen temperature intervals. Figure 6 presents Cole–Cole plots of *G″* versus *G′* at 0.1 and 1 Hz for iPP and samples containing 1.5 and 4 wt.% of HrGO. Being a semi-crystalline polymer, iPP appears to have a more complex thermorheological response, which is reflected by the formation of two peaks (see Figure 6), at lower and higher temperatures respectively in all available frequencies. The existence of these peaks can be associated with the *β* and $\alpha_{c}$ relaxation processes [61]. The formation of the first peak is related to the *β* relaxation process, which is attributed to segmental relaxation mechanisms in the amorphous region and has been related to glass transition. The second peak is related to the $\alpha_{c}$ relaxation process that is associated with relaxations arising from the crystalline phases of the polymer matrix [62,63]. The *α_c_*-relaxation in iPP can be considered as an exchange of stereodefects between amorphous and crystal phases. In our previous study, we noticed a marked suppression of the $\alpha_{c}$ relaxation as a function of filler loading from DMTA measurements on a single frequency [27]. This suppression is also evident in the Cole–Cole representation, especially for the highest filler loading (4 wt.%), something that can be associated with morphological changes due to the filler integration in the matrix [27,64].

### 3.3. Short-Term Tensile Properties

When discussing the mechanics of the HrGO–iPP nanocomposites, there are both factors that are expected to promote and factors that are expected to limit the efficient load transfer between the filler and the matrix. Firstly, we can be relatively confident that a certain amount of primary particles, i.e., rGO (~3–5 layers), are present in the system, without disregarding the existence of distorted fillers and further agglomerates that limit the overall aspect ratio and the access of the matrix to the all-important primary particles. Furthermore, we can expect that the overall amount of oxygen in the form of OH groups on the surface of the filler is fairly limited. Both of these assumptions are inferred from the existence of a low electrical percolation threshold and the relatively high values of electrical conductivity obtained previously [27]. Following the same reasoning, an efficient level of dispersion is to be expected, as confirmed by the morphological analysis. Both the high aspect ratio and superior dispersion would contribute favorably to the mechanical properties of the nanocomposites. However, distorted filers, that dominate the morphology, (see Figure 1c), and filler agglomerates are expected to be limiting factors for mechanical performance. Intimately connected to the dispersion level, the filler-matrix interfacial adhesion could be the determinant factor in the load transfer efficiency. Finally, in our previous work, we have shown that HrGO acts as a significant nucleation source in the iPP matrix [27], which can be another determinant factor of the efficiency of the filler-matrix load transfer. Even excluding additional effects of the nanofiller, nucleation of iPP causes an increase of Young’s modulus and decrease of impact properties [65,66]. To summarize, both favorable and detrimental factors could be considered for the mechanical properties of HrGO–iPP nanocomposites and this constitutes the framework of the discussion below.

**Figure 7:**
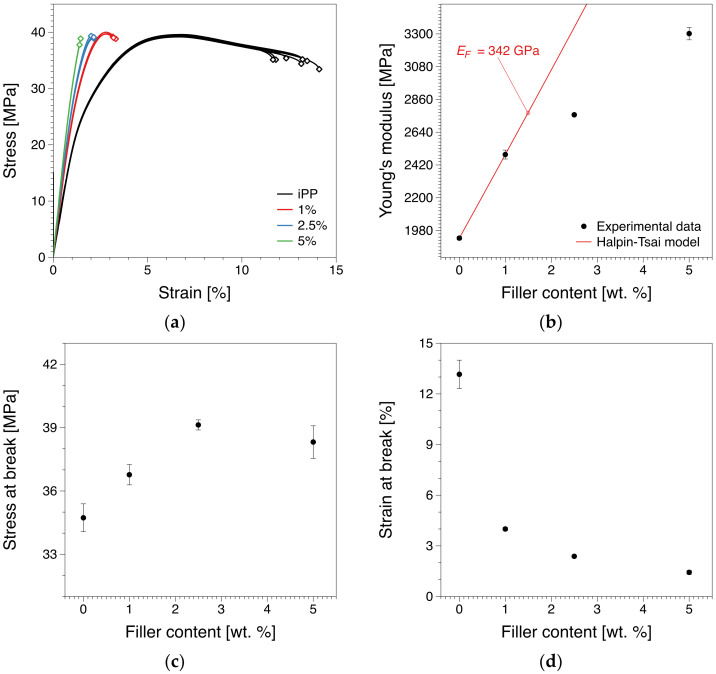
(**a**) Examples of stress-strain curves, (**b**) Young’s modulus experimental values compared with theoretical results computed using the Halpin-Tsai model, (**c**) stress at break and (**d**) strain at break values for the nanocomposites.

In Figure 7a examples of stress–strain curves for the different compositions are shown. The resulting Young’s moduli, stresses at break and strain at break are shown in Figure 7b–d. This can be associated with the extent of the network formation and is in qualitative agreement with previously reported results on similar materials [18,19,25,55,67,68,69,70]. From these findings, we can see a 30% increase in the modulus below the percolation threshold (~1 wt.%) [27], whereas the average increase above the electrical percolation threshold is a more moderate 15%. A mirrored behavior is observed in the strain at break decrease with increasing filler content. In contrast, the stress at break shows a slight monotonic increase until around 2.5 wt.%, after which the differences are negligible.

As indicated in Section 3.2.1, the addition of inorganic fillers into polymer matrices creates a network that leads to partial immobilization of the polymeric chains. In other words, the network structure can be seen as a form of physical crosslinking between the filler particles and the immobilized chains in their vicinity. This effect is further amplified by the extent of the filler network and the entangled nature of the polymer chains. During solidification, for a well dispersed system, the chains in the vicinity of the filler tend to be aligned with it, thus making lattice-matching the dominant force in the crystallization process [71]. This occurs due to the need for structural adjustments of the polymeric chains in the vicinity of the filler and the subsequent absorption on the graphitic surface. Consequently, the existence of the filler particle in the polymer matrix will enhance the heterogeneous nucleation, leading to increase in the degree of crystallinity or change in the crystalline form and the subsequent kinetics of the whole process [72]. These changes can improve the stiffness of the composite, something that is further accelerated by increasing the amount of filler added into the system and thus the potential for heterogeneous nucleation. In our case, we have previously seen that the filler leads to an increase in nucleation activity, especially below the electrical percolation that was followed with a significant change in the overall morphological characteristics, as seen through the type of crystals available to the system [27].

### 3.4. Micromechanical Modelling

As we have seen from the short-term tensile tests, the introduction of the filler leads to an increased stiffness of the nanocomposite. In order to evaluate the magnitude of this reinforcement, the well-established Halpin–Tsai model for randomly-oriented, reinforced polymers was used. The assumption of random orientation is the most viable option considering the sample preparation method [27]. In this we have assumed that the filler is acting like a rectangular solid fiber, in a manner similar to one suggested by Liu and co-workers [73]. In such a scheme, the elastic modulus of the nanocomposite, *E_C_*, can be estimated using the modified Halpin–Tsai equation [74,75].

(2) $$E_{C}=\frac{3}{8}\frac{1+\xi \eta_{L}V_{F}}{1-\eta_{L}V_{F}}E_{M}+\frac{5}{8}\frac{1+2\eta_{W}V_{F}}{1-\eta_{W}V_{F}}E_{M},$$

with

(3) $$\eta_{L}=\frac{\left(E_{F}/E_{M}\right)-1}{\left(E_{F}/E_{M}\right)+\xi }$$

(4) $$\eta_{W}=\frac{\left(E_{F}/E_{M}\right)-1}{\left(E_{F}/E_{M}\right)+2}$$

where *E_M_* and *E_F_* correspond to the Young’s modulus of the matrix and the filler respectively and *V_F_* is the filler volume fraction. The parameter *ξ* is associated with the geometry and boundary conditions of the filler, and for a rectangular shape can be expressed as [76].

(5) $$\xi =2\frac{\left(W+L\right)/2}{t}$$

where *W* is the average width of the filler (taken ~2–3 μm in our case), *L* the average length (4–10 μm), and *t* the average thickness (0.34–0.71 nm). The parameters used in this work are based on the characterization of the filler [27,77]. The filler volumes fraction can be determined from using the matrix (*ρ_M_*) and filler densities (*ρ_F_*) and the filler weight fraction (∅) as:

(6) $$V_{F}=\frac{\emptyset /\rho_{F}}{\emptyset /\rho_{F}+\left(1-\emptyset \right)/\rho_{M}}$$

where *V_F_* = 1 − *V_M_, V_M_* being the matrix volume fraction. The density of the matrix *ρ_M_* was taken as 0.945 g/cm^3^ and that of the filler *ρ_F_* as 2.2 g/cm^3^ per previously reported values [13]. A number of studies on the value of the Young’s modulus of reduced graphene oxide has been reported with values ranging from 200–400 GPa [78,79,80,81]. Here, it is worth noting that the values reported in the literature for the modulus are based on measurements on membranes or solution-based dispersed systems and on idealized theoretical predictions. In our case, we have a melt-mixed system where despite the significant level of dispersion, there is a significant level of filler distortion and some agglomerates do exist. Both are expected to lead to a decrease in the overall modulus response, compared to an ideal agglomerate-free primary particle system. Based on our experimental data, we have estimated that the filler used in this study is approximately 342 GPa in order for the Halpin–Tsai model to accurately reproduce the behavior we observe. For the estimation, concentrations above the electrical percolation threshold were not included. It should be noted that this is under the assumption of an ideal filler-matrix interface. In addition, the reported values for the mechanical properties of HrGO are dependent on the number of layers and the degree of coverage of the graphitic surface by OH groups [81]. In our case, we have a fairly low amount of OH groups on the graphitic surface, something that can be seen by the relatively high values of the electrical conductivity observed [27]. Furthermore, we must note that the observed value for the Young’s modulus corresponds to the response of all filler particles and agglomerates in the system. In terms of the Halpin–Tsai model, that means the value of the effective Young’s modulus will be associated with an average effective filler. Furthermore, the interface could also have a significant detrimental contribution, due to incompatibilities between the filler and matrix. These incompatibilities can be associated with the existence of OH groups on the filler surface that have the potential to create H-bonds with the chains in their vicinity, something that has been seen as the main reason behind changes in the glass transition temperatures [27]. In other words, we can be reasonably confident that we do get a reasonably good level of interfacial contact to some primary particles, as the theoretical Young’s modulus obtained is in the higher range of the reported values for reduced graphene oxide. However, larger spatial arrangements and agglomerates, as well as potential imperfect interfaces, are likely the limiting factors of the system compared to theoretical [82] and experimental reported values [83,84]. This confidence arises from the very low value of the electrical percolation threshold and the effect of the filler loading on the glass transition reported for this system [27] that indicates a very efficient level of dispersion where the polymer can get some degree of access to the primary particles.

Here we must note that our experimental data exhibits with a steep increase up to the percolation threshold ~1 wt.% [27,77]. Beyond that point the increase of the modulus is fairly restricted, something that can possibly be associated with the increased number of agglomerates as the filler content increases [17]. This is expected to have a detrimental effect on the load transfer between the filler and the matrix as the system is having reduced opportunities to get access to the primary particles, something that can be associated both with the electrical conductivity that reaches an almost saturation level [27] and the flow behavior of the material [77].

### 3.5. Creep

**Figure 8:**
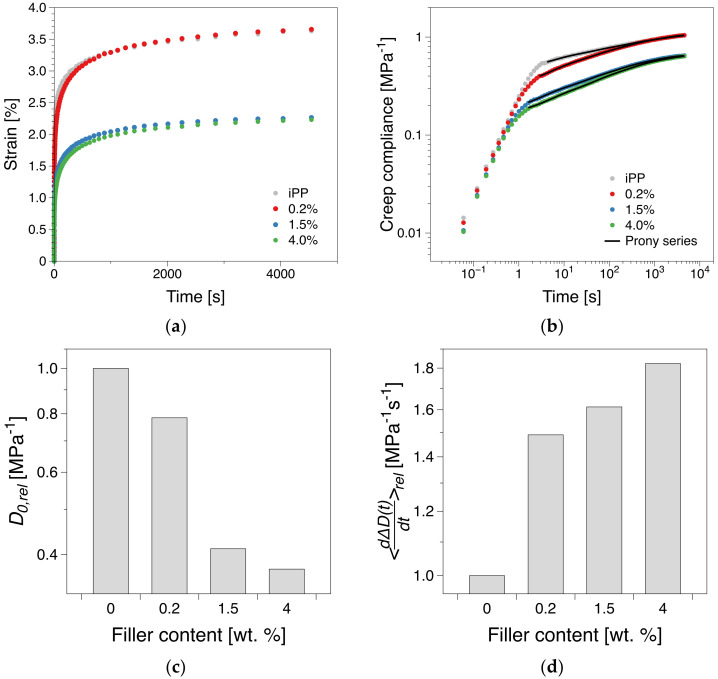
(**a**) Strain and (**b**) creep compliance dynamics during creep testing, and (**c**) the relative instantaneous compliance, $D_{0,rel}$, and (**d**) averaged transient rate of creep compliance ${<d\Delta D\left(t\right)/dt>}_{rel}$ dependence on filler concentration. A typical error for such determinations is 10%.

The variation of the strain output with time under constant stress conditions is shown in Figure 8a. The corresponding creep compliance, $D=\epsilon \left(t\right)/\sigma$, where $\epsilon \left(t\right)$ is the time-dependent strain and σ is the applied stress, is presented in Figure 8b. The transient creep compliance component, $\Delta D\left(t\right)$, does not obey a clear power-law dependence within the experimental time, however, it can be fitted by a five-mode Prony series of the form [30]:

(7) $$\Delta D\left(t\right)=D_{0}+\sum_{i=1}^{N}D_{i}\left(1-e^{-t/\tau_{i}}\right)$$

where $D_{0}$ is the instantaneous creep compliance. The obtained fit parameters are presented in the Supplementary Table S1. Figure 8c summarizes the tests in terms of relative instantaneous compliance $D_{0,rel}=D_{0}\left(\emptyset \right)/D_{0}\left(\emptyset =0\right)$ and relative averaged transient rate of creep compliance ${<d\Delta D\left(t\right)/dt>}_{rel}=<d\Delta D\left(t\right)/dt>\left(\emptyset \right)/<d\Delta D\left(t\right)/dt>\left(\emptyset =0\right)$, where ∅ is the filler concentration. Qualitative similarities with the short-term tensile test expected as both tests fall within the same load transfer scenario. However, in terms of experimental time scale relative to the material relaxation time, the instantantanous compliance more similar to the short-term tensile tests compared to the transient creep behavior. This distinction can be conjectured for our system as well. The relative instantanous compliance, $D_{0,rel}$, appears to behave qualitatively similar to the Young’s modulus and strain at break with the largest decrease (48%) between concentrations below (0.2 wt.%) and above (1.5 wt.%) the percolation threshold. However, in terms of averaged rate of creep compliance, ${<d\Delta D\left(t\right)/dt>}_{rel}$, the presence of the filler in the system makes a significant impact starting with the lowest filler loading (0.2 wt.%) with a 46% increase from the pure polymer, followed by a relatively moderate relative change for higher filler loadings (ca. 8 and 14%, respectively). This can be attributed to the transient behavior at the beginning of the secondary phase. Compared to the iPP, the filled samples exhibit a transitory behavior, approx. 1–10^2^ s, where there are likely adjustments in terms of filler-matrix interaction. This is indicated by the fact that iPP and 0.2 wt.% become virtually identical above 10^2^ s in terms of creep compliance, (Figure 8b).

### 3.6. Impact Strength

**Figure 9:**
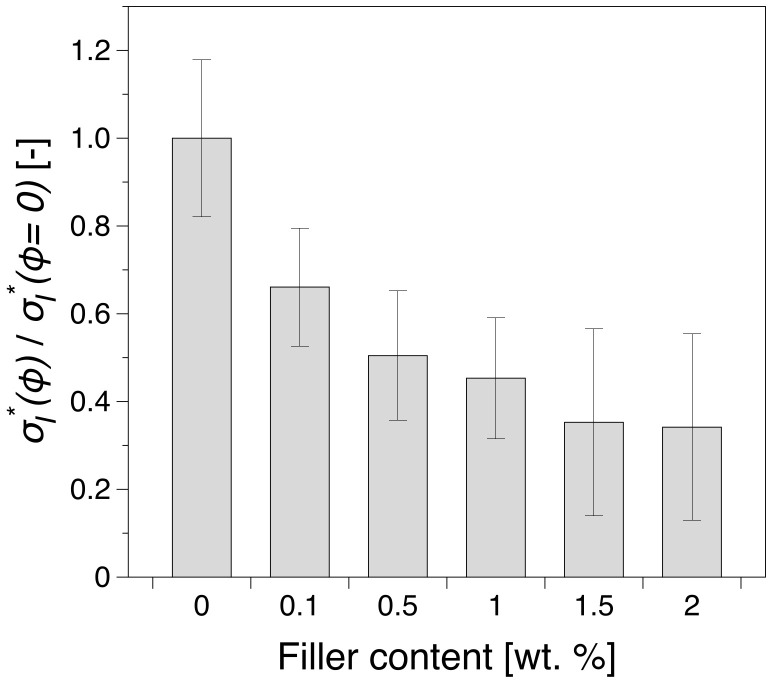
The relative impact strength, $\sigma_{I,rel}^{*}$, as a function of HrGO filler content.

Most polymer-based materials with good strength and stiffness exhibit brittle fracture at high strain rates. Therefore, the impact behavior of polymer composites is a key factor to evaluate their potential usage. Impact test quantifies the toughness or the impact strength of a material, which is defined as the ability of the material to absorb energy during plastic deformation. The absorbed energy is directly related to the toughness of the material. The relative impact strength $\sigma_{I,rel}^{*}=\sigma_{I}^{*}\left(\emptyset \right)/\sigma_{I}^{*}\left(\emptyset =0\right)$, where ∅ is the filler concentration, is shown in Figure 9 for all studied specimens.

It is evident that for lower filler loadings the influence of HrGO is greater than at higher concentrations. This indicates that the addition of the filler leads to a lower toughness of the nanocomposite. This behavior can be associated with the lower ductility observed for increased filler loading from the tensile tests, see Figure 6b,d. As the amount of filler in the system increases the extent of the network is also increasing, leading to higher values of modulus. This increase is more prominent below the percolation threshold, as in this region the network is not fully formed, whereas above the percolation threshold, the effect is fairly marginal. This increase in stiffness of the material can be correlated with an increase in brittleness observed in the impact measurements. Therefore, one can notice that the impact response which is imparted from HrGO is higher below electrical percolation, in contrast with loadings above the electrical percolation where the influence of HrGO is lower. This behavior can be associated to the level of dispersion and the existence of agglomerates that are present in the samples studied, especially those with higher filler concentration [27].

## 4. Summary and Conclusions

In this work, we have presented the mechanical properties of a novel, industrially relevant melt-mixed system of polypropylene (PP) and hierarchical reduced graphene oxide (HrGO). The filler used in this study allows for an efficient dispersion that leads to the creation of an extended three-dimensional extended structure [27]. The effects of this resulting structure were probed at different time-scales using several techniques, obtaining information regarding the dynamic, short-term tensile, creep and impact properties of the system.

The limitations in the applicability of the time–temperature superposition principle and in construction of the master curves for the PP-based nanocomposites has been outlined. The WLF method has been utilized to compute the activation energy of the nanocomposites showing an increase in energy with increase of the filler content. Cole–Cole curves have been shown to demonstrate the thermo-rheological character of the nanocomposites and probe their relaxation.

The mechanical properties of the iPP-HrGO nanocomposites, including short-term tensile and creep tests, are intimately related to a number of factors that could have both favorable and detrimental effects. This includes the filler aspect ratio, level of dispersion, interfacial strength, and crystallinity, as discussed at the beginning of the results section. Here, we have a system that exhibits very good levels of dispersion as seen by the low value of the electrical percolation threshold, and significant nucleation capability. Obviously, our filler is not in a state of pristine graphene sheet, rather a three-dimensional hierarchical structure, and the access to the primary particles, filler distortions due to compounding, and agglomerates are all aspects that need to be considered [27]. Judging from the low value of the electrical percolation threshold combined with increase in Young’s modulus, we can safely assume that there is a significant access of the matrix to the primary particles is present in the systems. Furthermore, due to the reasonably good dispersion and the morphological changes observed in our system, we can safely assume that the adhesion between the iPP and the filler could be significant, something that can be seen by the increasing trend of the Young’s modulus and even creep compliance behavior. As expected, with increasing stiffness comes an increase in brittleness, resulting in a lower strain at break and impact strength values as the filler content increases. More specifically, as the filler level increases, we can observe an increase in stiffness and brittleness, and a decrease in instantaneous creep compliance in all systems, while at higher filler loading above electrical percolation, the influence of the filler thereon is less pronounced. This can be associated with the change of the local spatial arrangements of chain in the vicinity of the filler that leads to partial immobilization. This in turn can lead to changes in the local dynamics of the system, something that can be seen by the changes observed in the glass transition temperature and the suppression of the *β* relaxation, especially above the electrical percolation threshold.

## Figures and Tables

**Table 1:** Summary of WLF equation parameters, Equation (1), from fitting the experimental data.

Sample	*T_g_* [K] [27]	$c_{1}^{r}$	$c_{2}^{r}\;[K]$	$c_{1}^{g}$	$c_{2}^{g}\;[K]$
iPP	273.15	24.6 ± 0.8	98 ± 4	33.0 ± 0.8	73 ± 4
1.5%	274.67	21.0 ± 0.7	82 ± 4	29.4 ± 0.7	58 ± 4
4.0%	274.76	50 ± 6	220 ± 30	56 ± 6	200 ± 30

## Supplementary Materials

The following are available online at https://www.mdpi.com/2073-4360/12/6/1309/s1, Supplementary information contains: Figure S1. Storage modulus at measured at different temperatures versus frequency for (a) iPP (b) 1.5 wt.% and (c) 4 wt.% samples. Table S1. Fit parameters of the Prony series. Table S2.
