# Supplementary material for: Mechanical Behavior of Melt-Mixed 3D Hierarchical Graphene/Polypropylene Nanocomposites
Source: Polymers (Basel). 2020 Jun 8;12(6):1309. doi: 10.3390/polym12061309 (PMC7361869; doi:10.3390/polym12061309)
Supplement: Supplementary file 1 [file polymers-12-01309-s001.pdf]

polymers

MDPI

Article

# Mechanical Behavior of Melt-Mixed 3D Hierarchical Graphene/Polypropylene Nanocomposites

**Karolina Gaska [1,*,†], Georgia C. Manika [1], Thomas Gkourmpis [2], Davide Tranchida [3], Antonis Gitsas [3] and Roland Kádár [1]**

1 Department of Industrial and Materials Science, Division of Engineering Materials, Chalmers University of Technology, SE-412 96 Gothenburg, Sweden; georgia.manika@chalmers.se (G.M.); roland.kadar@chalmers.se (R.K.)
2 Innovation & Technology, Borealis AB, SE-444 86 Stenungsund, Sweden; thomas.gkourmpis@borealisgroup.com
3 Innovation & Technology, Borealis Polyolefine GmbH, St.-Peter-Straße 25, 4021 Linz, Austria; davide.tranchida@borealisgroup.com (D.T.); antonis.gitsas@borealisgroup.com (A.G.)
* Correspondence: karolina.gaska@bristol.ac.uk
† Present address: Department of Aerospace Engineering, University of Bristol, Bristol BS8 1 TR, UK

Received: 12 May 2020; Accepted: 5 June 2020; Published: date

## Supplementary information

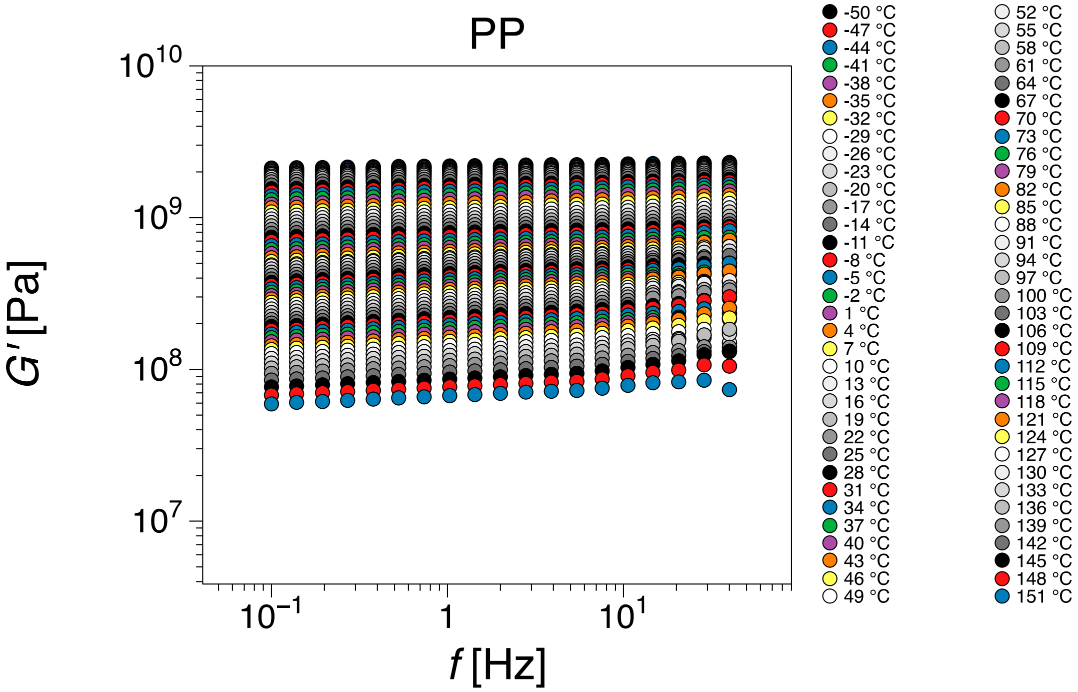


**(a)**

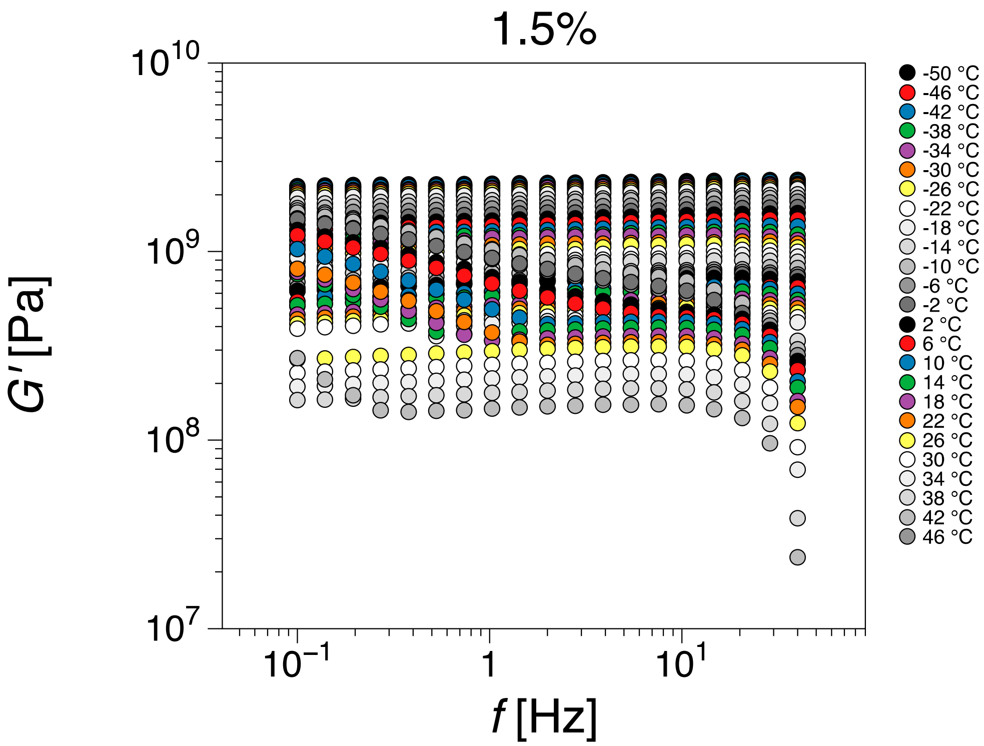


**(b)**

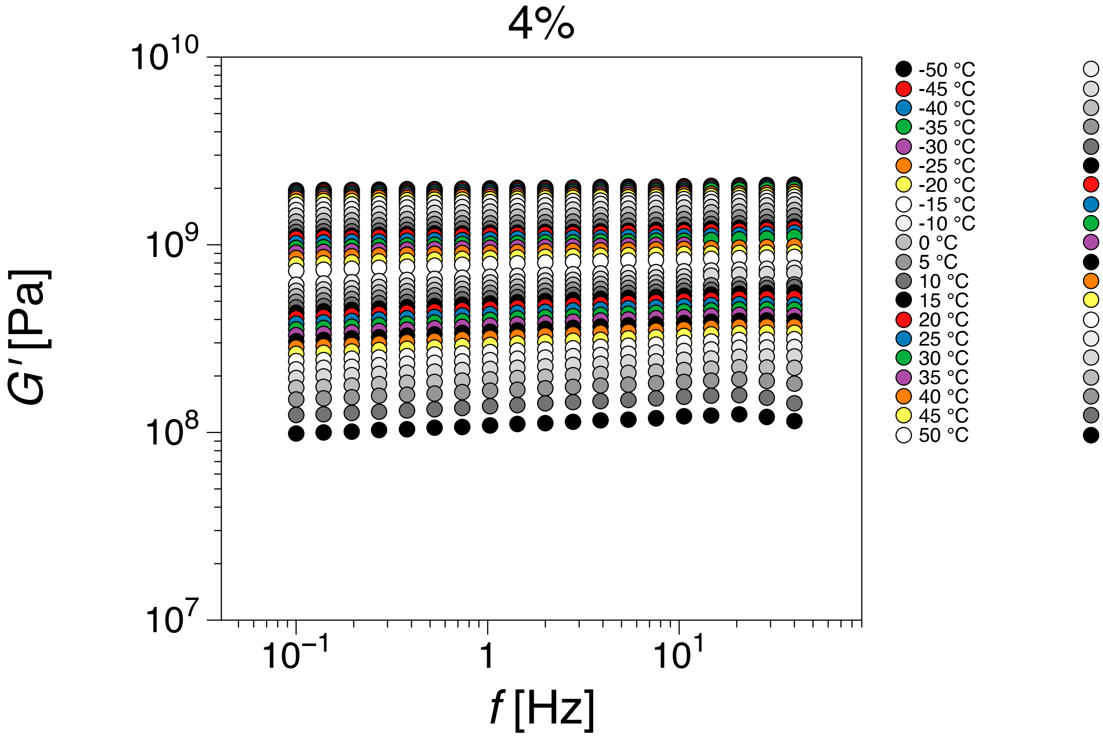


**(c)**

**Figure SI 1.** Storage modulus measured at different temperatures versus frequency for a) iPP b) 1.5 wt.% and c) 4wt.% samples.

**Table S1.** Fit parameters of the Prony series, Eq. 7, in Figure 8.

| | **iPP** | **0.2%** | **1.5%** | **4%** |
|---|---|---|---|---|
| $D_0$ [MPa$^{-1}$] | 0.45 | 0.31 | 0.17 | 0.16 |
| $D_1$, $\tau_1$ [MPa$^{-1}$],[s] | 0.16,5 | 0.18,5 | 0.13,173 | 0.09,5 |
| $D_2$, $\tau_2$ [MPa$^{-1}$],[s] | 0.18,2076 | 0.17,1588 | 0.11,26 | 0.12,40 |
| $D_3$, $\tau_3$ [MPa$^{-1}$],[s] | 0.14,254 | 0.2,36 | 0.1,3 | 0.14,1713 |
| $D_4$, $\tau_4$ [MPa$^{-1}$],[s] | 0.13,38 | 0.14,216 | 0.15,1248 | 0.15,254 |

© 2020 by the authors. Submitted for possible open access publication under the terms and conditions of the Creative Commons Attribution (CC BY) license (http://creativecommons.org/licenses/by/4.0/).
